# Protein phosphorylation networks in spargana of *Spirometra erinaceieuropaei* revealed by phosphoproteomic analysis

**DOI:** 10.1186/s13071-020-04119-w

**Published:** 2020-05-13

**Authors:** Wei Liu, Hailin Tang, Asmaa M. I. Abuzeid, Lei Tan, Aibing Wang, Xueping Wan, Haoji Zhang, Yisong Liu, Guoqing Li

**Affiliations:** 1 Guangdong Provincial Zoonosis Prevention and Control Key Laboratory, College of Veterinary Medicine, South China Agricultural University, Guangzhou, 510642 People’s Republic of China; 2 College of Veterinary Medicine, Hunan Agricultural University, Changsha, 410128 Hunan People’s Republic of China; 3 The Key Laboratory of Animal Vaccine & Protein Engineering, Changsha, 410128 Hunan People’s Republic of China; 4 State Key Laboratory of Oncology in South China, Collaborative Innovation Center for Cancer Medicine, Sun Yat-Sen University Cancer Center, Guangzhou, 510060 Guangdong Province People’s Republic of China; 5 Department of Physiology, Medical College of Georgia at Augusta University, Augusta, GA 30912 USA; 6 College of Life Science and Engineering, Foshan University, Foshan, 528225 Guangdong Province People’s Republic of China

**Keywords:** *Spirometra erinaceieuropaei*, Spargana, IMAC, Mass spectrometry, Phosphoproteome

## Abstract

**Background:**

Sparganosis caused by *Spirometra erinaceieuropaei* spargana is a zoonotic parasitic infection that has been reported in many countries, including China, Japan, Thailand and Korea, as well as European countries and the USA. The biological and clinical significance of the parasite have previously been reported. Although the genomic and transcriptomic analysis of *S. erinaceieuropaei* provided insightful views about the development and pathogenesis of this species, little knowledge has been acquired in terms of post-translational regulation that is essential for parasite growth, development and reproduction. Here, we performed site-specific phosphoproteomic profiling, with an aim to obtain primary information about the global phosphorylation status of spargana.

**Results:**

A total of 3228 phosphopeptides and 3461 phosphorylation sites were identified in 1758 spargana proteins. The annotated phosphoproteins were involved in a variety of biological pathways, including cellular (28%), metabolic (20%) and single-organism (17%) processes. The functional enrichment of phosphopeptides by Gene Ontology analysis indicated that most spargana phosphoproteins were related to the cytoskeleton cellular compartment, signaling molecular function, and a variety of biological processes, including a molecular function regulator, guanyl-nucleotide exchange factor activity, protein kinase activities, and calcium ion binding. The highly enriched pathways of phosphorylation proteins include the phosphatidylinositol signaling system, phagosome, endocytosis, inositol phosphate metabolism, terpenoid backbone biosynthesis, and peroxisome. Domain analysis identified an EF-hand domain and pleckstrin homology domain among the key domains.

**Conclusions:**

To our knowledge, this study performed the first global phosphoproteomic analysis of *S. erinaceieuropaei*. The dataset reported herein provides a valuable resource for future studies on the signaling pathways of this important zoonotic parasite.
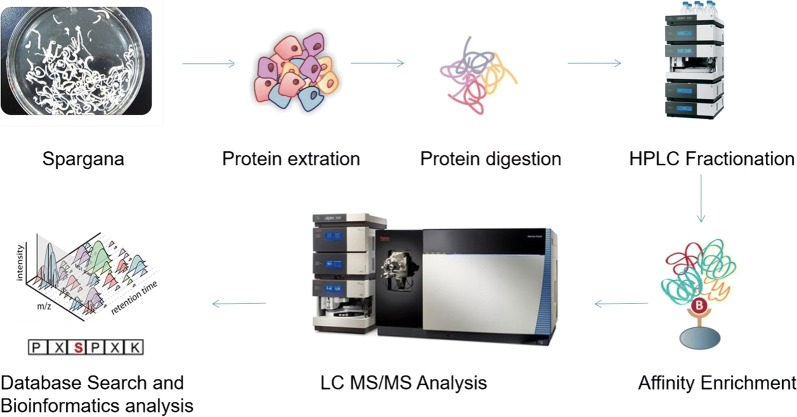

## Background

*Spirometra erinaceieuropaei* (syns. *Spirometra mansoni*, *S. erinacei*) belongs to the order Diphyllobothriidea in the class Cestoda [1]. Its motile larvae, plerocercoids (spargana), cause sparganosis in humans. Sparganosis has been reported in China, Japan, Thailand and Korea, as well as in European countries and the USA [2]. Humans can be infected by ingesting raw or undercooked infected intermediate hosts, including frogs, snakes and fish, as well as drinking water contaminated with infected copepods or using frog flesh as a poultice on eyes or wounds, a common practice in traditional Chinese medicine [3]. Once inside their host, spargana are able to reach many sites, including eyes, abdominal cavity, subcutaneous tissue and brain, causing a variety of symptoms depending on their location, such as blindness, headaches, convulsions and even death [4, 5].

The genome of *S. erinaceieuropaei* was reported in 2014, providing valuable genomic information for this previously uncharacterized zoonotic tapeworm [6]. The expanded gene families in *S. erinacei* sparganum genome include several genes associated with post-translational modifications of proteins. The functions of these proteins included protein folding and were found primarily within the serine/threonine kinase families, as well as other kinases [7]. A major reversible post-translational modification in eukaryotes is the phosphorylation of proteins at specific enzymes, such as serine and tyrosine residues, that play important roles in the regulation of signaling pathways in many cellular processes [8]. Protein phosphorylation is reversibly controlled by networks of phosphatases and kinases. As a result, protein kinases alter the functions of other proteins by adding phosphate groups [9]. The addition of phosphate groups can change the stability, activity, interactions, and localization of the individual proteins. Therefore, abnormal phosphorylation is often associated with diseases, including diabetes, neurodegeneration, and even cancer [10]. Recently, phosphoproteomic techniques based on phosphopeptide enrichment methods, such as metal oxide affinity chromatography and immobilized metal affinity chromatography (IMAC) [11, 12], in combination with mass spectrometry (MS), have been used to investigate large-scale protein phosphorylation profiles in many organisms. As a result, phosphoproteomes have not only been analyzed in humans [13], but also in parasites, including *Schistosoma japonicum*, *Leishmania* sp., and *Toxoplasma gondii*. In *S. japonicum*, protein phosphorylation also plays an important role in the growth, development, and reproduction of the parasite, for which the tyrosine protein kinase (TK) and protein kinase C (PKC) are the representatives [14]. The activity of LmjAQP1 in *Leishmania* sp. is regulated by mitogen-activated protein kinase 2 (MAPK2). The phosphorylation of LmjAQP1 protein can reduce its metabolic rate and prolong its activity [15]. Calcium-dependent phosphorylation of myosin A in *T. gondii* plays an important role in the movement of the parasite and its invasion to host cells [16]. In spite of this, few proteins related to phosphorylation have been identified in spargana of *Spirometra mansoni* [17]. Furthermore, no data are available regarding the phosphorylation sites, the kinases and phosphatases involved, or the cellular processes targeted by these post-translational modifications.

In this study, the proteins in *S. erinaceieuropaei* spargana were digested with enzymes. The resulting phosphopeptides were analyzed using a combination of IMAC with MS to elucidate the *in vivo* phosphorylation events in *S. erinaceieuropaei* spargana. A total of 3461 phosphorylation sites (p-sites) were found in 1758 spargana proteins. These findings provide an insight into the functions regulated by protein phosphorylation in *S. erinaceieuropaei*.

## Methods

### Isolation of spargana and preparation of protein extracts

Spargana were isolated from the subcutaneous and muscular tissues of the snake *Zaocys dhumnades* (Wushao snake) from the Xiangxiang City, Hunan Province, China, according to the protocol described by Muller et al. [18], and then washed thoroughly with phosphate-buffered saline (PBS, pH 7.4). The plerocercoids were 10–15 cm long. A pool of plerocercoids (*n* = 10) from one snake were used to identify more phosphoproteins. Three biological replicates (*n* = 10 each) were performed to improve the reliability of identifying phosphorylation sites, including two replicates with the soluble protein fraction and one with the insoluble protein fraction. The specimens were frozen in liquid nitrogen for 1 h and then stored at − 80 °C until further use.

The frozen samples were ground into powder and placed in a centrifuge tube (5 ml). Subsequently, four volumes of lysis buffer comprised of 8 M urea and 1% protease inhibitor cocktail (Abcam, Cambridge, UK) were added and then sonicated three times on ice. Centrifugation was performed at 12,000×*g* at 4 °C for 10 min to remove the remaining debris. Finally, a bicinchoninic acid (BCA) assay was conducted to determine the protein concentration in the resulting supernatant.

### Digestion of proteins

The tryptic digestion of the proteins was carried out as described by Ren et al. [19], with some modifications. Briefly, the protein solution was reduced with 5 mM dithiothreitol at 56 °C for 30 min, followed by alkylation with 11 mM iodoacetamide at room temperature in the dark for 15 min, then dissolved into urea solution with the addition of 100 mM NH_4_HCO_3_. Finally, the first and second rounds of digestion were conducted in a solution with a 1:50 ratio of trypsin-to-protein mass overnight, and a 1:100 ratio of trypsin-to-protein mass for 4 h, respectively.

### Fractionation by HPLC

High pH reversed-phase HPLC was performed to fragment the tryptic peptides using a Thermo Betasil C18 column (5 μm particles, 10 mm ID, and 250 mm length) (Thermo Fisher Scientific, Waltham, MA, USA). The peptides applied to an 8–32% gradient of acetonitrile (pH 9.0) were initially separated into 60 fractions in 60 min. The resulting peptides were then combined into 7 fractions, followed by evaporation in a vacuum centrifuge.

### Enrichment of phosphopeptides

The enriched phosphopeptides were dissolved in NETN buffer (consisting of 0.5% NP-40, 100 mM NaCl, 50 mM Tris-HCl and 1 mM EDTA, pH 8.0) and incubated with pre-washed IMAC antibody beads (CST, Danvers, USA) with gentle shaking at 4 °C overnight. The beads were then washed with NETN buffer four times and with H_2_O twice. Then, 0.1% trifluoroacetic acid was used to elute the bound peptides from the beads and the eluted vacuum-dried fractions were combined. Liquid chromatography-tandem mass spectrometry (LC-MS/MS) analysis was performed on the resulting peptides, desalted with Millipore C18 ZipTips (Sigma-Aldrich, Shanghai, China). All the experiments were conducted according to the manufacturer’s instructions. The results were analyzed using two-dimensional LC-MS/MS [20].

### Analysis of NanoLC-MS/MS

The tryptic peptides dissolved in 0.1% formic acid (solvent A) were directly loaded onto an in-house made reversed-phase analytical column (15 cm length, 75 μm internal diameter). The solvent B (0.1% formic acid in 98% acetonitrile) gradients were gradually increased as follows: 6% to 23% over 26 min, then 23% to 35% over 8 min, and finally up to 80% over 3 min. The gradient was held at 80% for an additional 3 min at a constant flow rate of 400 nl/min using an EASY-nLC 1000 ultra-performance liquid chromatography (UPLC) system (Thermo Fisher Scientific).

The peptides were subjected to nano-spray ionization (NSI) followed by tandem mass spectrometry (MS/MS) in Q Exactive™ Plus (Thermo Fisher Scientific) coupled with the online UPLC system. A 350–1800 m/z scan range and a 2.0 kV electrospray voltage was used for the full scan at a resolution of 70,000 to detect the intact peptides. A nominal collision energy (NCE) of 28% was used to select the peptides for MS/MS. A resolution of 17,500 in the Orbitrap was used to detect the fragments. A data-dependent procedure that switched between one MS scan and 20 MS/MS scans was initiated for the top 20 precursor ions above a 5.0E3 threshold ion count in the MS survey scan with 15.0 s dynamic exclusion, while automatic gain control (AGC) was set at 5E4.

### Identification of phosphopeptides

The mass spectrometry data were retrieved with Maxquant v1.5.2.8. The parameter settings for retrieval were set by comparing the transcriptome database generated in this study with a reverse library search to identify the false discovery rate (FDR) resulting from random matching. A common contamination library was compared with the database to eliminate the influence of contaminated proteins on the identification results. The enzyme digestion mode was specified as trypsin/P, and the number of missing cut-points was set at 2. The minimum length and the maximum modification number of peptide segments were set as 7 and 5 amino acid residues, respectively. The first and main search were customized to 20 ppm and 5 ppm, respectively, while the mass error tolerance of secondary debris ions was 0.02 Da. Cysteine alkylation was set as a fixed modification reference, while methionine oxidation, n-terminal acetylation of proteins, and phosphorylation of serine, threonine and tyrosine were incorporated into alterable modification references. The FDR for both protein and peptide-spectrum match (PSM) identification was 1%.

### Analysis of motifs

The web-based *motif-x* program (http://motif-x.med.harvard.edu/motif-x.html) was used to analyze models of protein sequences in terms of amino acids at specific positions of modify-21-mers (10 amino acids upstream and downstream of the site) in all the protein sequences [21]. The database protein sequences were used as background parameters, with the default settings used for the other parameters. When the number of peptides in the sequence was greater than 20 and the statistical test *P*-value was less than 0.000001, a characteristic sequence was regarded as a motif of the modified peptide.

### Functional annotation and classification of phosphoproteome

BLAST analysis was performed using Blast2GO [22]. Phosphoprotein enrichment of function was performed using BINGO according to Gene Ontology (GO) [23] and the plug-ins in the Cytoscape platform [24]. The GO terms, which are significantly enriched in the phosphoproteins, were compared to the group of “both non-phosphoproteins and phosphoproteins”. Based on the selected phosphoproteins, the hypergeometric distribution of these phosphoproteins to a specific branch(s) in the GO classification was calculated. The GO analysis would return a hypothetical *P*-value for each GO term in which phosphoproteins exist, and a small *P*-value would indicate the enrichment of the differential genes in GO. Fisher’s exact test was employed to test the enrichment of the differentially modified proteins against all identified proteins. For each category of phosphoproteins, the InterPro database (http://www.ebi.ac.uk/interpro/) was inspected, and Fisher’s exact test was utilized. A corrected *P*-value < 0.05 for the protein domains was considered significant.

### Pathway analysis of phosphoproteins

The Kyoto Encyclopedia of Genes and Genomes (KEGG) database (https://www.genome.jp/kegg/) was used to identify the enriched pathways. The classified pathways were shown in the hierarchical categories according to the data from the KEGG website.

### Enrichment-based clustering

For further hierarchical clustering based on different protein functional classifications (e.g. GO, domain, pathway, complex), we first collated all the categories obtained after enrichment, along with their *P*-values, and filtered for the categories that were at least enriched in one of the clusters with a *P*-value < 0.05. This filtered *P*-value matrix was transformed by the function x = − log_10_ (*P*-value). Finally, these x-values were z-transformed for each functional category. These z-scores were then clustered using one-way hierarchical clustering (Euclidean distance, average linkage clustering) in Genesis. Cluster membership was visualized by a heat map using the “heatmap.2” function from the *gplots* package in R.

## Results and discussion

### Establishment of the spargana phosphoproteome

The development of a parasitic infection in a host occurs *via* signals exchanged between the host and the parasite [7]. Post-translational modifications, such as protein phosphorylation, may play an important role in the transmission of these signals, thereby playing a critical role in the process of host infection. Previous studies have found that the phosphorylation of important amino acid sites can result in the modification of phosphorylated proteins, leading to the inhibition of protein kinase and phosphatase activity. This disordered protein phosphorylation process can have serious repercussions on the physiological state of the host, allowing for parasitic growth and invasion, as well as altering signaling pathways. As such, the in-depth study of protein phosphorylation is of great significance to improve our understanding of the role of protein phosphorylation in parasite growth and development, and could help to provide an experimental basis for the screening of candidate vaccine molecules and new drug targets against parasites.

In this study, to the best of our knowledge, we report the first global phosphoproteomic dataset of *S. erinaceieuropaei* spargana. In total, 3228 phosphopeptides and 3461 phosphorylation sites were identified from 1758 spargana proteins. Details on the identified phosphopeptides are provided in Additional file 1: Table S1 and Additional file 2: Table S2, including the identification algorithm scores and PTM scores. We performed three biological replicates to improve the reliability of phosphorylation site identification, including two replicates with the soluble protein faction and another with the insoluble protein fraction. These data can be downloaded from iProX (http://www.iprox.org).

**Fig. 1:**
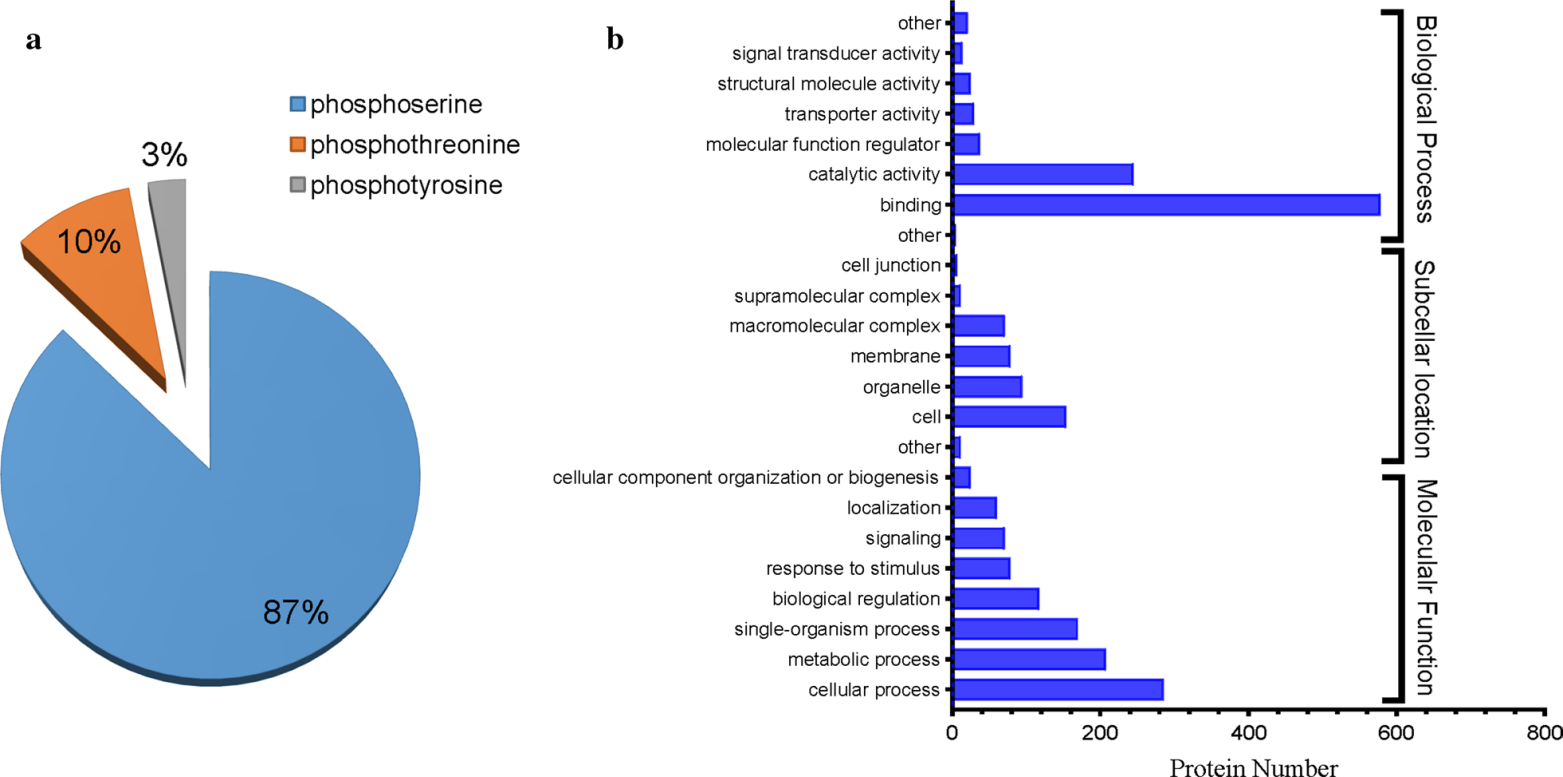
General description of *S. erinaceieuropaei* spargana phosphoproteome data. **a** Pie chart representation of the distribution of identified phosphorylation sites. **b** GO term distribution of spargana phosphoproteins in three categories. GO annotation and categorization were performed using Blast2GO

Among the 3461 phosphorylation sites identified, phosphoserine comprised the highest proportion (87%), followed by phosphothreonine (10%), and phosphotyrosine (3%) (Fig. 1a). The modification ratio of phosphoserine, phosphothreonine and phosphotyrosine found in this study was similar to that described previously in other organisms [25].

Using the online automatic annotation tool Blast2GO, a functional annotation was conducted to characterize the functional distribution of the 1758 identified phosphoproteins [22]. After BLAST analysis, annotation and mapping, we found that 45% (799/1758) of the identified phosphoproteins were annotated with at least one GO node in each of the three GO categories. The distribution of the three categories of GO terms for the sparganum phosphoproteins is shown in Fig. 1b and Additional file 3: Table S3.

**Fig. 2:**
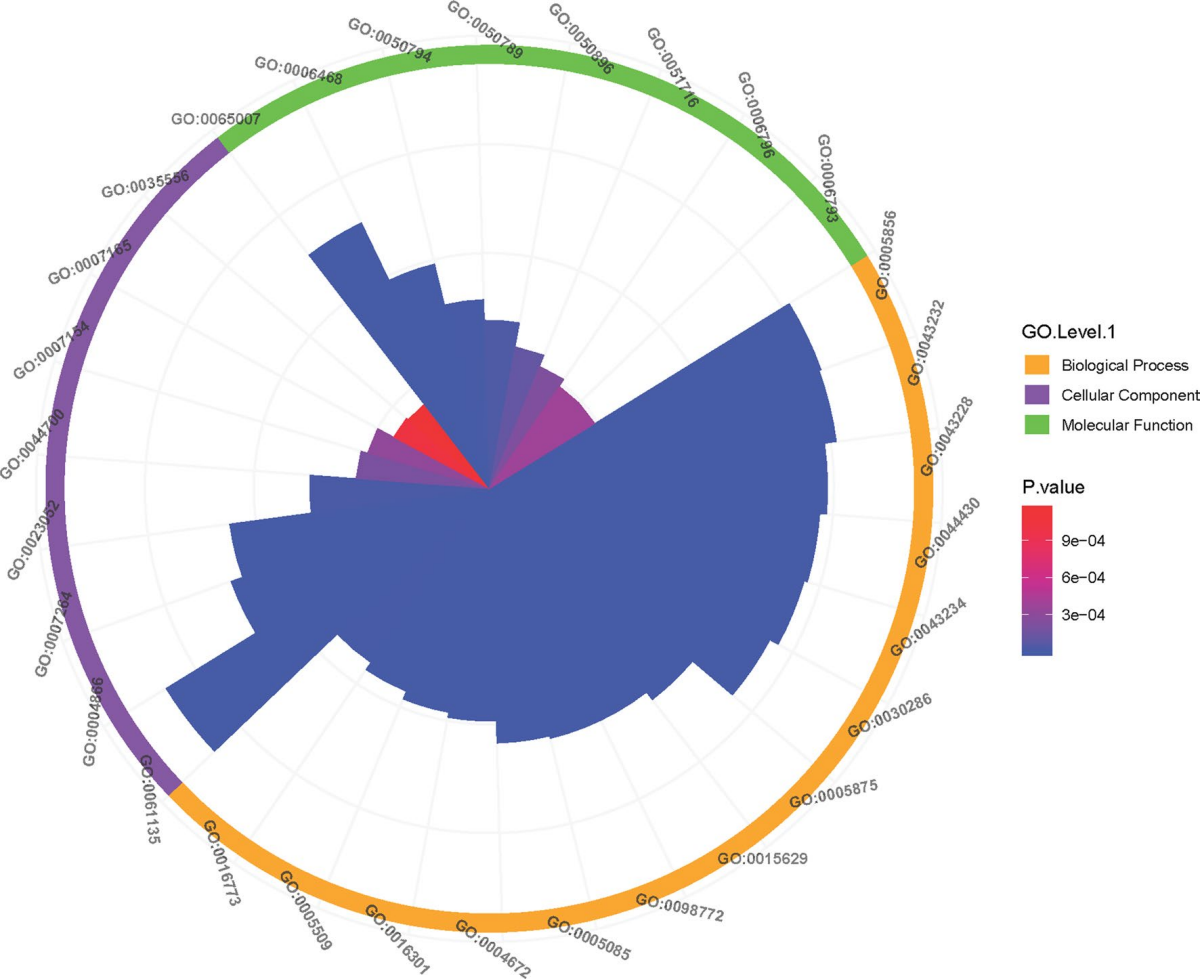
GO enrichment analysis of the phosphoproteins. The GO analysis of the phosphoproteins falls into the following GO categories: cellular component, biological process and molecular function

In the category of biological processes, the annotated phosphoproteins participated in a wide variety of biological processes, including cellular, metabolic, and single-organism processes, in response to biological regulation, stimulus, signaling, cellular component organization, localization and biogenesis (Fig. 1b), which are closely related to the pleiotropic nature of protein phosphorylation. Regarding the molecular function GO category, the highest proportion of phosphoproteins was associated with their binding (61%), followed by the different activities, such as catalytic activity, transporter activity, structural molecule activity and signal transducer activity (Fig. 2). Meanwhile, Wolfpsort, a novel version of PSORT/PSORT II for predicting eukaryotic sequences, was employed as a predictor of subcellular localization (Additional file 4: Table S4, Additional file 5: Figure S1). We found that the subcellular localization of the phosphoproteins was consistent with the GO analysis, with over 40% of the phosphorylated proteins found in the nucleus, then the cytoplasm (21%), extracellular matrix (13%), mitochondria (10%) and plasma membrane (8%). Interestingly, other organelles (e.g. peroxisome and Golgi apparatus) accounted for only a small portion of the phosphorylated proteins.

### Characterization of amino acid residues around the phosphorylation sites

The occurrence of each amino acid in phospho-21-mers among the spargana was compared to determine the composition of the amino acid residues flanking the phosphorylation sites. Phosphosites were determined using the *motif-x* algorithm, with 25 motifs categorized as a result. The motifs identified included 21 phosphoserine motifs and 4 phosphothreonine motifs. The phosphotyrosine motifs were undetectable, which may be explained by the low abundance of tyrosine-phosphorylated peptides.

**Fig. 3:**
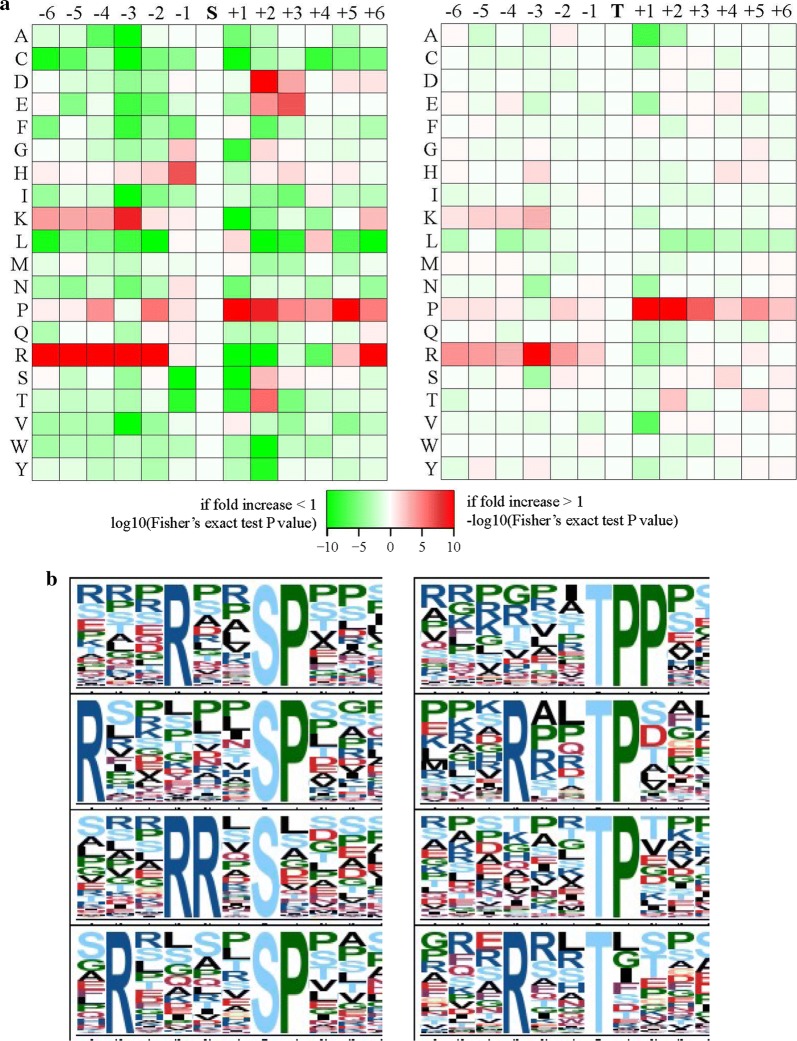
Phosphorylation motif enrichment in *S. erinaceieuropaei* spargana. **a** Motif enrichment heat maps of the upstream and downstream amino acids of all identified phosphorylation modification sites (red indicates that the amino acid is significantly enriched near the modification site; green indicates that the amino acid is significantly reduced near the modification site). **b** Phosphopeptides were analyzed using *motif-x* webserver. The top 4 enriched motifs in phosphoserine (left) and phosphothreonine (right)

Phosphoserine motifs containing the “R…SP…” (fold increase = 11.01), “R…SP…” (fold increase = 9.56), “..…RR.S…” (fold increase = 10.07), and “R…S …” (fold increase = 9.96) were the top-four motifs phosphorylated by kinases (Fig. 3a, Additional file 6: Table S5). The top-four phosphothreonine motifs were “…TPP…” (fold increase = 27.35), “…R…TP…” (fold increase = 28.95), “…TP…” (fold increase = 6.7), and “…R…T…” (fold increase = 5.37). We then carried out NetworKIN analysis to determine the probable upstream kinase of the motifs enriched by phosphoproteins identified in the retinoblastoma [26]. In this study, we identified “…SP…”, a proline-directed sequence, which is the recognition site of the cyclin-dependent kinase of mitogen-activated protein kinase (MAPK) cells [27]. Furthermore, the identified “…R…S…” is a basic motif-type sequence, as well as the recognition site for Ca^2+^/Cam-dependent protein kinase (Additional file 6: Table S5). In general, a protein kinase differentiates certain motifs *via* its substrate [28].

### Analysis of functional enrichment for phosphoproteins

**Fig. 4:**
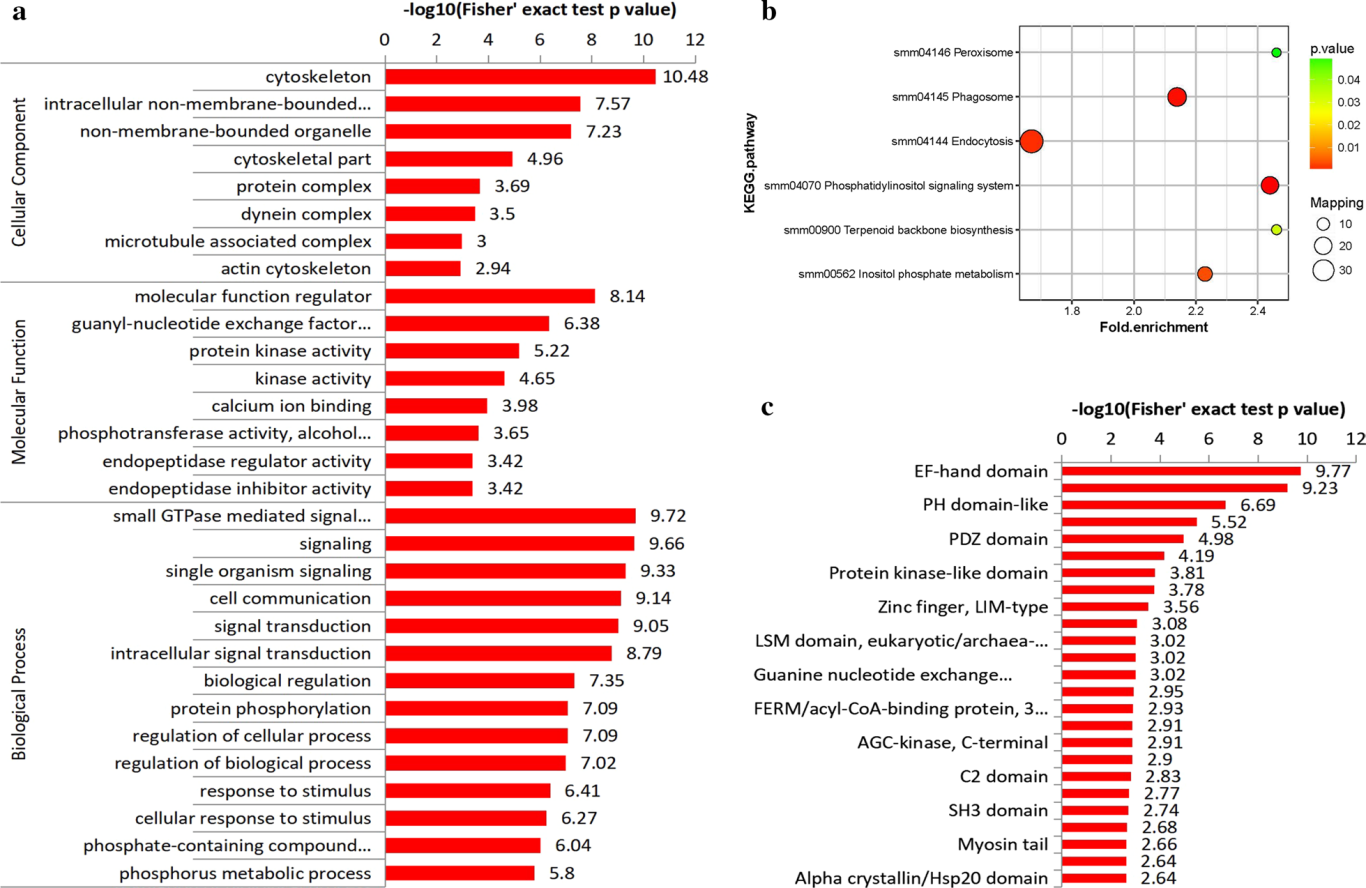
Enrichment analysis of GO (**a**), KEGG (**b**) and domains analysis (**c**) of *S. erinaceieuropaei* spargana phosphoproteome. Cluster membership were visualized by a heat map using the “heatmap.2” function in the *gplots* package in R

GO analysis of phosphoproteins was performed to determine the potential functional processes involved (Fig. 3b, Additional file 7: Table S6). Protein cellular components were found to be significantly increased in two areas: cytoskeleton (GO: 0005856; GO: 0044430; GO: 0015629) and complex (GO: 0043234; GO: 0030286; GO: 0005875). The majority of phosphoproteins were related to the cytoskeleton, suggesting their importance in the spargana. The top 5 enriched GO terms related to the molecular function category were all related to signaling (GO: 0007264; GO: 0023052; GO: 0044700; GO: 0007154; GO: 0007165). The majority of the biological processes involved were related to certain fundamental routes, including a molecular function regulator (GO: 0098772), guanyl-nucleotide exchange factor activity (GO: 0005085), protein kinase activity (GO: 0004672), kinase activity (GO: 0016301), and calcium ion binding (GO: 0005509) (Fig. 4a, Additional file 7: Table S6).

### Enriched pathways of sparganum phosphoproteins

To determine whether the phosphoproteins identified play a role in any biological or signaling pathways, the identified phosphoproteins were analyzed against the KEGG database (Fig. 4b, Additional file 8: Table S7). The phosphatidylinositol signaling system (environmental information processing; signal transduction) was found to be the most enriched pathway of the phosphorylation proteins (Additional file 5: Figure S2). Phosphatidylinositol (PtdIn) is a small lipid molecule containing an inositol ring and two fatty acid chains connected by a glycerol backbone, which confirmed that PtdIns will be permitted to reside in the cytoplasmic face of the cellular membranes [29]. PtdIns is phosphorylated by the host lipid kinases at the 3, 4, and/or 5 hydroxyl positions of the inositol ring. A series of phosphatidylinositol monophosphates (PI3P, PI4P and PI5P), diphosphates [PI(3,4)P2, PI(3,5)P2, PI(4,5)P2], and a triphosphate [PI(3,4,5)P3] were produced, which collectively named the phosphoinositides [30].

Additionally, the phagosome (Additional file 5: Figure S3) and endocytosis (Additional file 5: Figure S4) pathways (cellular processes: transport and catabolism) showed enrichment in our system. Phagocytosis, the process of cells engulfing relatively large particles, is the central pathway for the tissue transformation, inflammation, and defense against infectious agents [31]. After formation, nascent phagosomes progressively acquire digestive characteristics. Toxic products are released by the fusion of phagosomes and lysosomes to kill the pathogens and degrade them into inactivated fragments [32]. Endocytosis is a cellular mechanism involving the removal of ligands, nutrients, plasma membrane (PM) proteins, and lipids from the cell surface and their transport into the intracellular space [33]. Transmembrane proteins subjected to clathrin-dependent endocytosis (CDE) carry certain sequences in their cytoplasmic domains that bind to the adaptor-related protein complexes (APs), thereby allowing for their rapid removal from the PM [34]. In addition to APs and clathrin, there are many accessory proteins, including dynamin. Cargoes are sorted into distinct destinations depending on the protein composition and incorporated into the endosome membrane. For example, nutrient receptors are recycled back into the PM. Ubiquitylated membrane proteins, such as activated growth-factor receptors, are sorted into intraluminal vesicles and finally reach the lysosome lumen *via* multivesicular endosomes (MVEs). Different mechanisms of clathrin-independent endocytosis (CIE) occur depending upon the cell type and the cargo [35].

The inositol phosphate metabolism (carbohydrate metabolism) was also among the enriched pathways of spargana phosphopeptides in our study (Additional file 5: Figure S5) and may contribute to providing energy to this species of parasite. Similarly, the terpenoid backbone biosynthesis pathway (metabolism of terpenoids and polyketides) was enriched in the spargana phosphoproteome (Additional file 5: Figure S6). The biosynthesis of these molecules forms the precursors of sterols (C30) and carotenoids (C40). The MEP/DOXP pathway is absent in higher animals and fungi, while the MEP/DOXP and mevalonate pathways co-exist in separate cellular compartments in green plants. The mevalonate pathway, occurring in the cytosol, results in the production of triterpenes, sterols and sesquiterpenes.

The InterPro program was used to classify the phosphoproteins into different families and predict the presence of domains and important sites (Fig. 4c, Additional file 9: Table S8). The EF-hand domain was found to be significantly enriched, resulting in domain enrichment. Many calcium-binding proteins belong to the same evolutionary family and share an EF-hand domain [36]. This type of domain consists of a 12-residue loop flanked on both sides by alpha-helical domain and the calcium ion is arranged in a pentagonal bipyramidal configuration [37]. EF-hands tend to exist in pairs or high copy numbers and the binding are found at positions 1, 3, 5, 7, 9 and 12 [38].

The pleckstrin homology (PH) domains represent the 11th most common domain in the human proteome. They are best known for their ability to bind phosphoinositides with a high affinity and specificity, although it is now clear that less than 10% of all PH domains share this property [39]. The results of this domain, as well as the pathway enrichment, both indicate the potential importance of the phosphoinositides pathway in the sparganum.

### Comparison with phosphoproteomes of other parasites

According to the spargana phosphoproteome, there was no up- or downregulation, except for a relative level of enrichment. We compared the phosphoproteins and peptides of *S. japonicum*, *P. falciparum* and *T. gondii* from the phosphoproteome database in an attempt to identify any possible connections between the phosphoproteome and the biological processes of the parasite. MAP kinase (MAPK), which was previously found to be related to the invasion of *Tetrahymena thermophila* [25], was found in the spargana (18 related phosphoproteins were identified) (Table 1). We also found several tyrosine kinases (17 related phosphoproteins) that correlated with *Schistosoma*, associated with the development of vitellarium and ovaries in females [14]. The protein kinase C&A, c-Jun N-terminal kinases and cyclin-dependent kinase, associated with the host-parasite interaction and reproduction, were also identified. The relationship between different parasites could help us to characterize our profile further [15, 40].

**Table 1:** Function related phosphoproteins family

Related enzyme	Phosphoproteins identified in spargana	Main function	Related species
MAP kinase (MAPK)	18	Invasion of parasites	*Tetrahymena thermophila*
Tyrosine kinase (TK)	17	TK was paired with *Schistosoma* and associated with the development of the vitellarium and ovaries in females	*Schistosoma japonicum*
Protein kinase C (PKC) and A	10	The phosphorylation of PKC can inhibit the transformation of cercaria	*Schistosoma japonicum*
c-Jun N-terminal kinases (JNK)	6	The protein is abundant in the adult and can participate in host-parasite interaction	*Plasmodium falciparum*
Cyclin-dependent kinase (CDK)	4	CDK play an important role in the asexual reproduction of *Plasmodium* parasites	*Plasmodium falciparum*

In *T. gondii*, a zoonotic parasite, the phosphoprotein eIF (eukaryotic initiation factor) plays an important role in the growth and development of the parasite; however, it was not found during our analysis. This may have resulted from the time point of our sample, or due to the use of different parasite species [41].

## Conclusions

To the best of our knowledge, this is the first study to analyze the phosphoproteome of *S. erinaceieuropaei* spargana. Based on this analysis, several phosphoproteins were identified and their functions were predicted. The phosphoproteins identified were found to be related to changes in many system processes. Furthermore, the integrated analysis of the phosphoproteome was used to predict the biological functions of many unannotated proteins, providing an overview of protein phosphorylation in *S. erinaceieuropaei* spargana. In future studies, we will focus on the phosphoproteomics of this parasite at different life-cycle stages.

## Supplementary information

**Additional file 1: Table S1.** Detailed list of phosphorylation sites detected in sparganum.

**Additional file 2: Table S2.** Detailed list of phosphorylated peptides detected in sparganum.

**Additional file 3: Table S3.** Detailed list of GO terms classification.

**Additional file 4: Table S4.** Subcellular classification of phosphorylated proteins.

**Additional file 5: Figure S1.** Subcelluar location of phosphorylated proteins. **Figure S2.** Phosphatidylinositol signaling system (Environmental Information Processing; Signal transduction). **Figure S3**. Phagosome (Cellular Processes; Transport and catabolism). **Figure S4.** Endocytosis (Cellular Processes; Transport and catabolism). **Figure S5.** Inositol phosphate metabolism (Carbohydrate metabolism). **Figure S6**. Terpenoid backbone biosynthesis (Metabolism of terpenoids and polyketides).

**Additional file 6: Table S5.** Functional enrichment of phosphoproteins in the GO analysis.

**Additional file 7: Table S6.** Functional enrichment of phosphoproteins in the KEGG pathway database.

**Additional file 8: Table S7.** Functional enrichment of phosphoproteins in protein domain.

**Additional file 9: Table S8.** Detailed list of *motif-x* analysis results.

## Data Availability

All data supporting the findings of this article are included in the main article and its additional files. The mass spectrometry proteomics data have been deposited at the ProteomeXchange Consortium *via* the PRIDE partner repository with the dataset identifier PXD015753.

## Abbreviations

IMAC: immobilized metal affinity chromatography
MS: mass spectrometry
BCA: bicinchoninic acid
LC-MS/MS: liquid chromatography-tandem mass spectrometry
UPLC: ultra-performance liquid chromatography
NSI: nano-spray-ionization
NCE: nominal collision energy
AGC: automatic gain control
PSM: peptide-spectrum match
GO: Gene Ontology
KEGG: Kyoto Encyclopedia of Genes and Genomes
