# Supplementary material for: Protein phosphorylation networks in spargana of Spirometra erinaceieuropaei revealed by phosphoproteomic analysis
Source: Parasit Vectors. 2020 May 13;13:248. doi: 10.1186/s13071-020-04119-w (PMC7218563; doi:10.1186/s13071-020-04119-w)
Supplement: Supplementary file 5 — Additional file 5: Figure S1. Subcelluar location of phosphorylated proteins. Figure S2. Phosphatidylinositol signaling system (Environmental Information Processing; Signal transduction). Figure S3. Phagosome (Cellular Processes; Transport and catabolism). Figure S4. Endocytosis (Cellular Processes; Transport and catabolism). Figure S5. Inositol phosphate metabolism (Carbohydrate metabolism). Figure S6. Terpenoid backbone biosynthesis (Metabolism of terpenoids and polyketides). [file 13071_2020_4119_MOESM5_ESM.docx]

**Additional file 5: Figure S1.** Subcellular location of phosphorylated proteins.


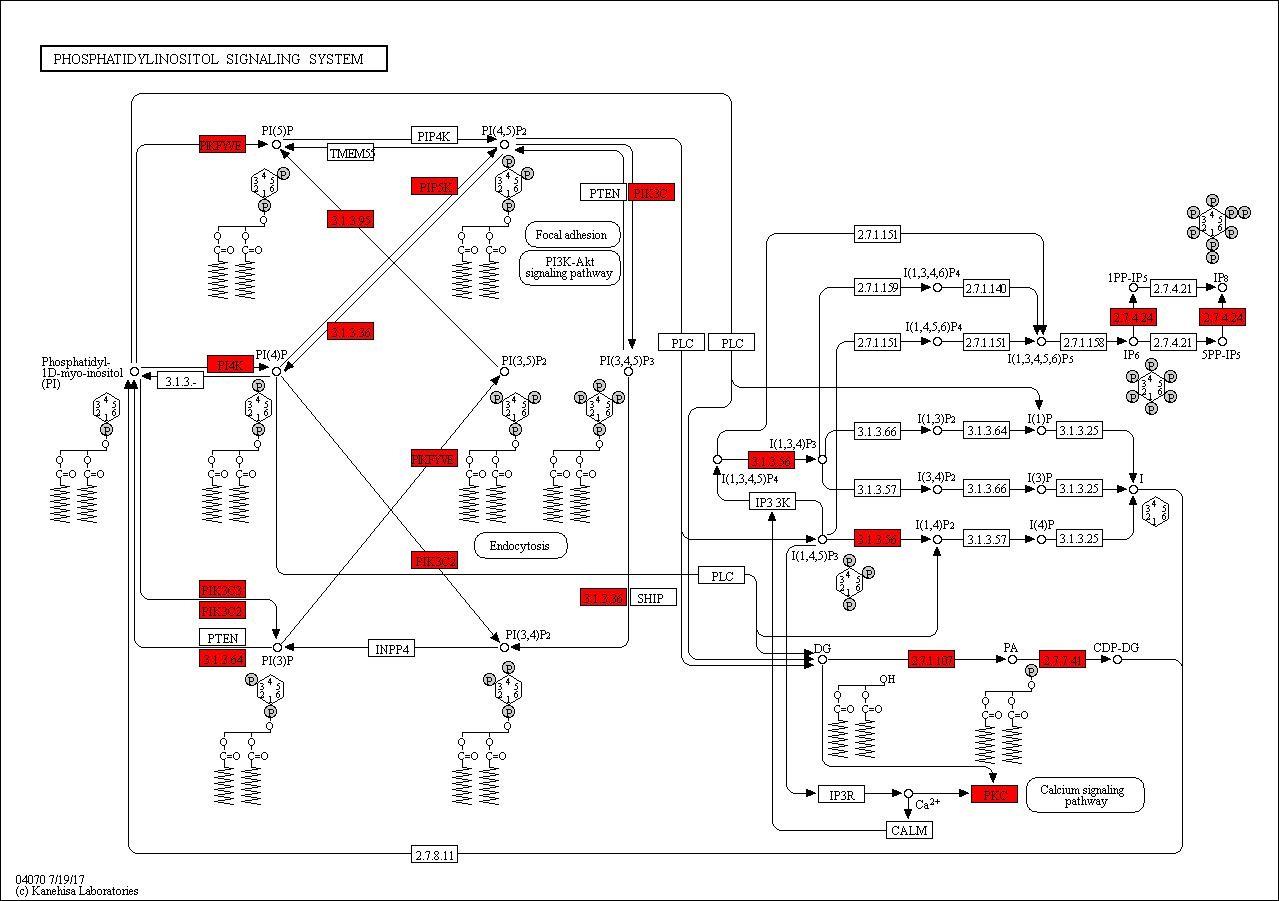


**Additional file 5: Figure S2.** Phosphatidylinositol signaling system (Environmental Information Processing; Signal transduction).


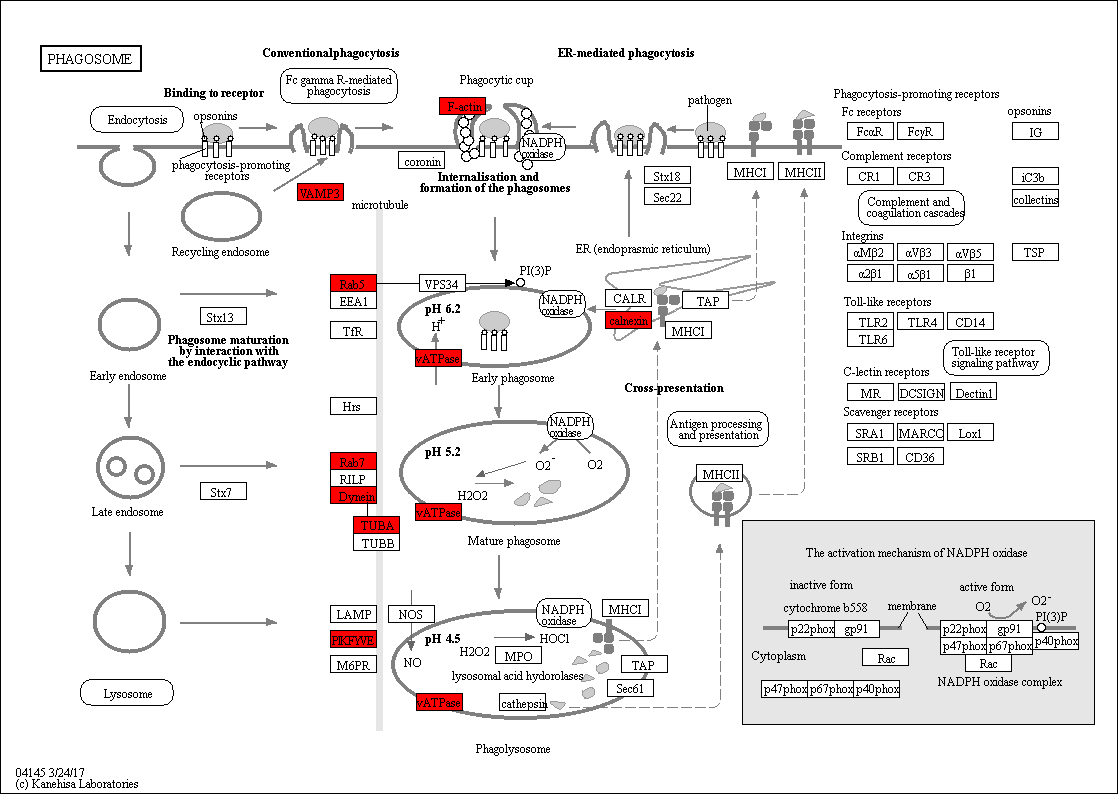


**Additional file 5: Figure S3**. Phagosome (Cellular Processes; Transport and catabolism).


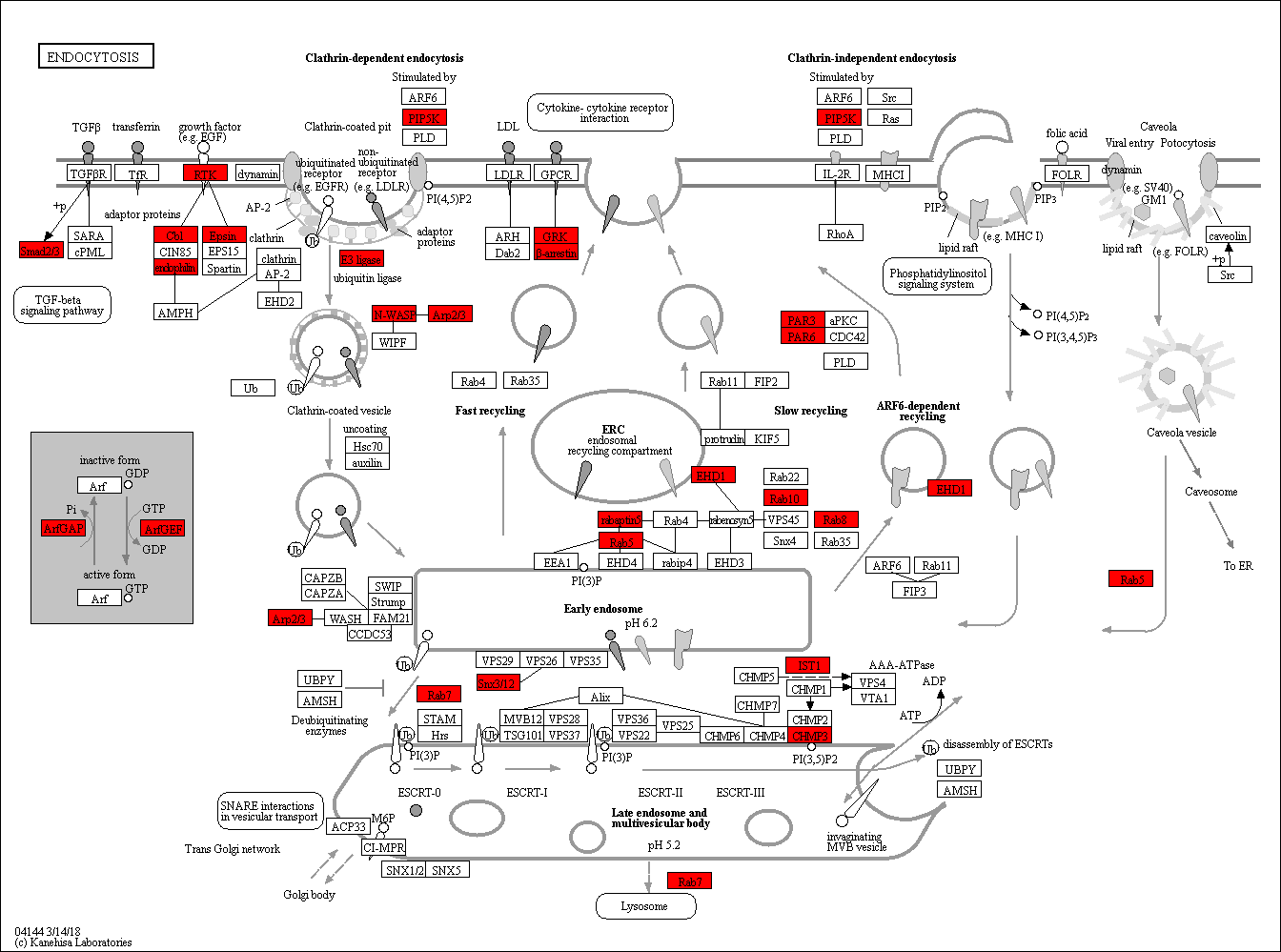


**Additional file 5: Figure S4.** Endocytosis (Cellular Processes; Transport and catabolism).


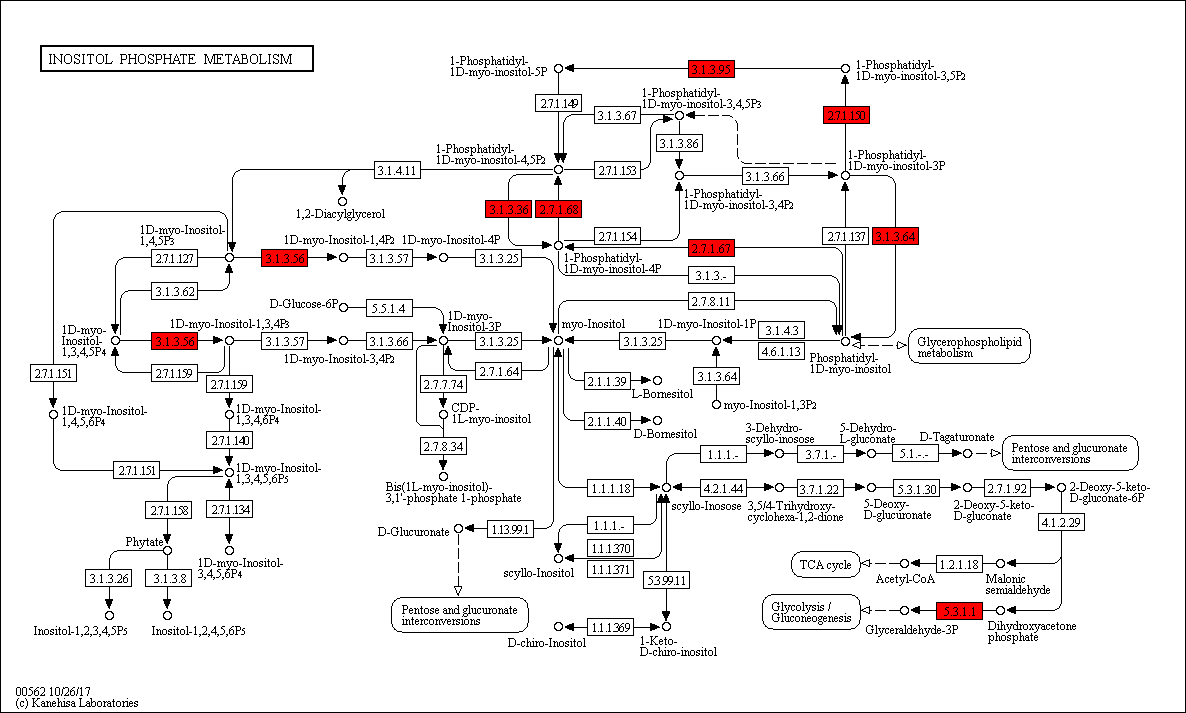


**Additional file 5: Figure S5.** Inositol phosphate metabolism (Carbohydrate metabolism).


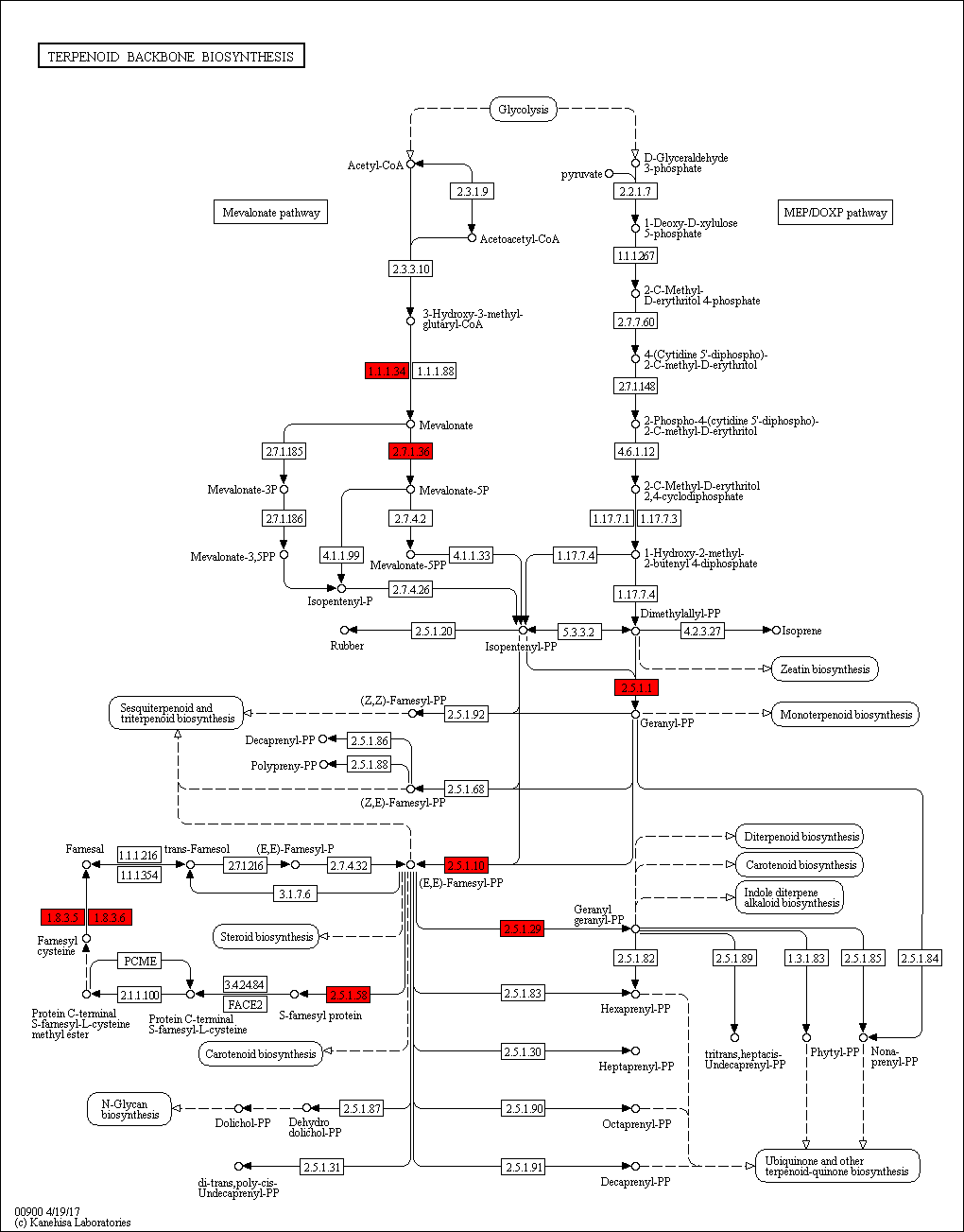


**Additional file 5: Figure S6**. Terpenoid backbone biosynthesis (Metabolism of terpenoids and polyketides).
